# Grape-Derived Polyphenols Improve Aging-Related Endothelial Dysfunction in Rat Mesenteric Artery: Role of Oxidative Stress and the Angiotensin System

**DOI:** 10.1371/journal.pone.0032039

**Published:** 2012-02-27

**Authors:** Noureddine Idris Khodja, Thierry Chataigneau, Cyril Auger, Valérie B. Schini-Kerth

**Affiliations:** UMR CNRS 7213 - Laboratoire de Biophotonique et Pharmacologie, Université de Strasbourg, Faculté de Pharmacie, Illkirch, France; Heart Center Munich, Germany

## Abstract

Aging is characterized by the development of an endothelial dysfunction, which affects both the nitric oxide (NO)- and the endothelium-derived hyperpolarizing factor (EDHF)-mediated relaxations, associated with vascular oxidative stress and the activation of the angiotensin system. This study investigated whether red wine polyphenols (RWPs), antioxidants and potent stimulators of NO- and EDHF-mediated relaxations improve aging-related endothelial dysfunction, and, if so, examined the underlying mechanism. Mesenteric artery reactivity was determined in organ chambers, vascular oxidative stress by dihydroethidine and MitoSOX staining, and expression of target proteins by immunohistochemical staining. Control young rats (16 weeks) received solvent (ethanol, 3% v/v), and middle-aged rats (46 weeks) either solvent or RWPs (100 mg/kg/day) in the drinking water. The acetylcholine-induced endothelium-dependent NO component was slightly reduced whereas the EDHF component was markedly blunted in rings of middle-aged rats compared to young rats. The endothelial dysfunction was associated with oxidative stress, an upregulation of angiotensin II and AT1 receptors and a down-regulation of SK_Ca_, IK_Ca_, and angiotensin converting enzyme. Intake of RWPs for either one or two weeks improved the NO and the EDHF components of the relaxation, and normalized oxidative stress, the expression of SK_Ca_, IK_Ca_ and the components of the angiotensin system. The protective effect of the 2-week RWPs treatment persisted for one and two weeks following stopping intake of RWPs. Thus, intake of RWPs caused a persistent improvement of the endothelial function, particularly the EDHF component, in middle-aged rats and this effect seems to involve the normalization of the expression of SK_Ca_, IK_Ca_ and the angiotensin system.

## Introduction

The endothelium is a key regulator of vascular homeostasis mostly through the release of several potent vasoactive factors that control vascular tone, blood fluidity, inflammation and smooth muscle cell proliferation. The endothelium-derived relaxing factors, which promote vascular protection, include nitric oxide (NO), prostacyclin, and endothelium-derived hyperpolarizing factor (EDHF) [1]. The importance of the EDHF phenomenon increases as the arterial diameter decreases and, hence, it has been suggested to play a significant role in the regulation of peripheral vascular resistance [2]. In the mesenteric artery as well as in several other types of arteries, EDHF-mediated responses involve the activation of endothelial SK_Ca_ and IK_Ca_ channels (small and intermediate conductance Ca^2+^-activated K^+^ channels, respectively) inducing hyperpolarization of the endothelium which is thereafter transmitted, in part, to the underlying vascular smooth cells via myo-endothelial gap junctions with subsequent relaxation [3].

Vascular aging is associated with the development of an endothelial dysfunction, which may contribute to the initiation and development of major cardiovascular diseases such as atherosclerosis and hypertension. Aging-related endothelial dysfunction has been described in different vascular beds including the human brachial artery [4], the rat aorta [5], the rat carotid artery [6] and the rat perfused mesenteric bed [7]. The aging-related endothelial dysfunction often involves a decreased NO- [8] and EDHF-mediated relaxations [9], [10], and also, in some blood vessels, the development of endothelium-dependent contractile responses [11]. It is associated also with an excessive vascular formation of reactive oxygen species (ROS), in particular superoxide anions, which, in turn, can inactivate NO [12], [13]. Potential sources of ROS in old arteries include NADPH oxidase [12], mitochondrial respiration chain [14], xanthine oxidase [15], and uncoupled endothelial NO synthase [16]. Although the mechanism underlying the aging-related oxidative stress is unclear, recent evidence suggests a role for the angiotensin system. Indeed, both an angiotensin-converting enzyme inhibitor and an AT1 receptor antagonist have been shown to prevent the aging-related endothelial dysfunction [10], [17]. In addition, angiotensin II, which is a potent inducer of vascular oxidative stress via the AT1 receptor-dependent upregulation of NADPH oxidase [18], has also been shown to induce a severe inhibition of EDHF-mediated relaxations in the mesenteric artery [19].

Numerous vascular reactivity studies indicate that several polyphenol-rich sources such as red wine polyphenols and green tea catechins are potent inducers of both NO- and EDHF-mediated endothelium-dependent relaxations [20], [21], [22]. In addition, chronic intake of red wine polyphenols improved the angiotensin II-induced hypertension and endothelial dysfunction in rats [18]. The beneficial effect of red wine polyphenols involves their ability to prevent vascular oxidative stress, in part, by reducing the expression of NADPH oxidase [18]. Moreover, chronic intake of red wine polyphenols by young rats prevented aging-related endothelial dysfunction in the mesenteric artery and decline in physical performance [23]. Therefore, the aim of the present study was to determine whether established aging-related endothelial dysfunction can also be improved by the intake of red wine polyphenols, and, if so, to determine the time-course of the induction of the vascular protective effect and its removal after stopping intake of red wine polyphenols, as well as the role of oxidative stress and the angiotensin system.

## Results

### Aging is associated with blunted NO- and EDHF-mediated relaxations in the mesenteric artery

**Figure 1:**
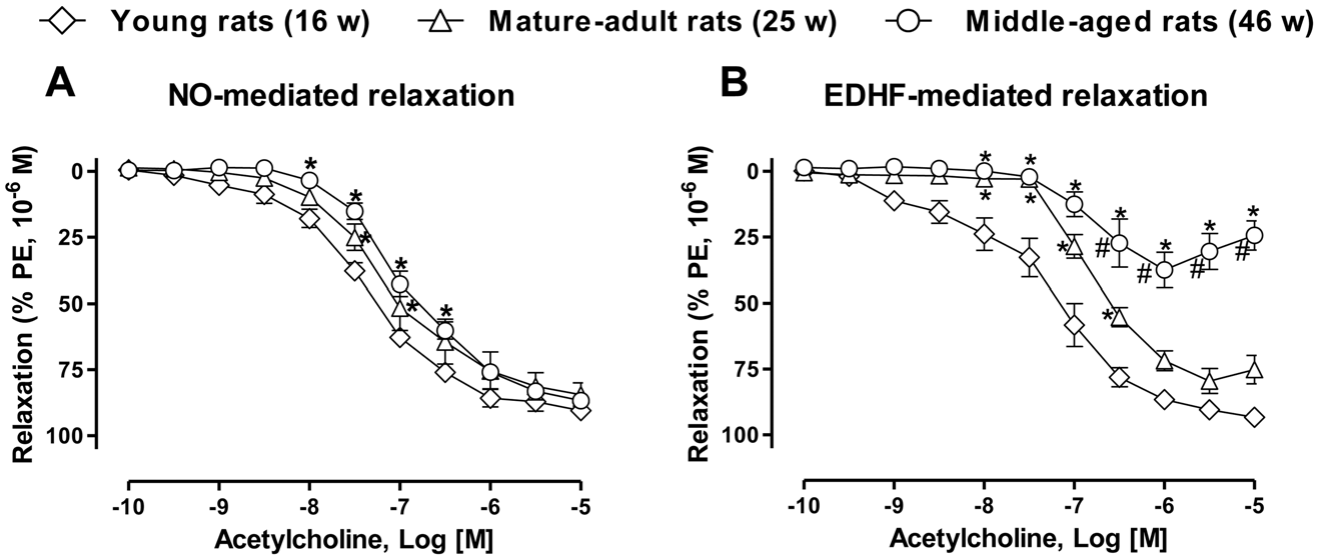
Acetylcholine-induced NO- and EDHF-mediated relaxations decrease with increasing age. Mesenteric artery rings from young (16-week old), mature-adult (25-week old) and middle-aged (46-week old) rats were contracted with phenylephrine (1 µM) in the presence of indomethacin (10 µM, to inhibit the formation of prostanoids) and (a) charybdotoxin (CTX, 100 nM) plus apamin (APA, 100 nM) to inhibit the participation of EDHF, or (b) N^ω^-nitro-L-arginine (L-NA, 300 µM) to rule out the formation of NO before a concentration-relaxation curve to acetylcholine was constructed. Results are shown as mean ± SEM of 5 to 6 different rats. *P<0.05 indicates a significant difference versus young rats and ^#^ *P*<0.05 indicates a significant difference versus mature-adult rats.

In mesenteric artery rings with endothelium, acetylcholine-induced NO-mediated relaxations (determined in the presence of indomethacin, and charybdotoxin plus apamin to prevent the formation of vasoactive prostanoids and EDHF, respectively) were slightly but significantly reduced in mature-adult (25-week old) and middle-aged (46-week old) rats compared to young (16-week old) rats (Figure 1a). In addition, acetylcholine-induced EDHF-mediated relaxations (determined in the presence of indomethacin and L-NA to prevent the formation of vasoactive prostanoids and NO, respectively) were reduced significantly to some extent in mature-adult rats and markedly blunted in middle-aged rats in comparison to young rats (Figure 1b). In contrast, relaxations in mesenteric artery rings without endothelium to the exogenous donor of NO, sodium nitroprusside (EC_50_ were 3.0±0.5 nM, 1.8±0.9 nM, 2.5±0.9 nM for 16, 25 and 46-week old rats, respectively; n = 5–6) and the ATP-sensitive K^+^ channel opener, levcromakalim (EC_50_ were 118.3±35.4 nM, 274.2±95.2 nM, 154.9±52.7 nM for 16-, 25- and 46 week-old rats, respectively; n = 5–6) were similar in the three groups of age.

### Intake of RWPs improves NO- and EDHF-mediated relaxations in middle-aged rats

**Figure 2:**
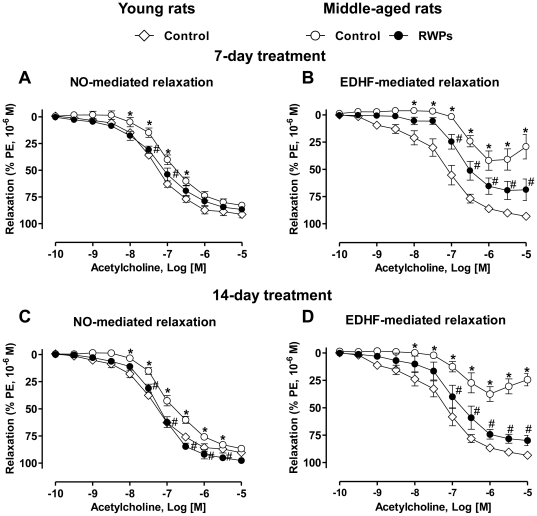
Intake of RWPs improves blunted NO- and EDHF-mediated relaxations in the mesenteric artery of middle-aged rats. Young (16-week old) and middle-aged (46-week old) control rats received solvent (ethanol, 3% v/v) in the drinking water and middle-aged treated rats received RWPs (100 mg/kg/day) for either 7 (a, b) or 14 days before sacrifice (c, d). NO-mediated relaxations were determined in rings contracted with phenylephrine (1 µM) in the presence of indomethacin (10 µM) and charybdotoxin (CTX, 100 nM) plus apamin (APA, 100 nM) to inhibit the participation of prostanoids and EDHF, respectively (a, c). EDHF-mediated relaxations were determined in the presence of indomethacin (10 µM) and N^ω^-nitro-L-arginine (L-NA, 300 µM) to rule out the participation of prostanoids and NO, respectively (b, d). Results are shown as mean ± SEM of 5 to 6 different rats. *P<0.05 indicates a significant difference versus the young rat control group and ^#^ *P*<0.05 a significant difference versus the middle-aged rat control group.

Intake of RWPs (100 mg/kg/day) in the drinking water for either 7 or 14 days improved slightly but significantly NO-mediated relaxations and markedly EDHF-mediated relaxations in the mesenteric artery of middle-aged rats (Figure 2). The 7- and 14-day RWPs treatments did affect neither relaxations to sodium nitroprusside nor those to levcromakalim in mesenteric artery rings without endothelium (data not shown).

### Persistent protective effect of the RWPs treatment on NO- and EDHF-mediated relaxations in middle-aged rats

**Figure 3:**
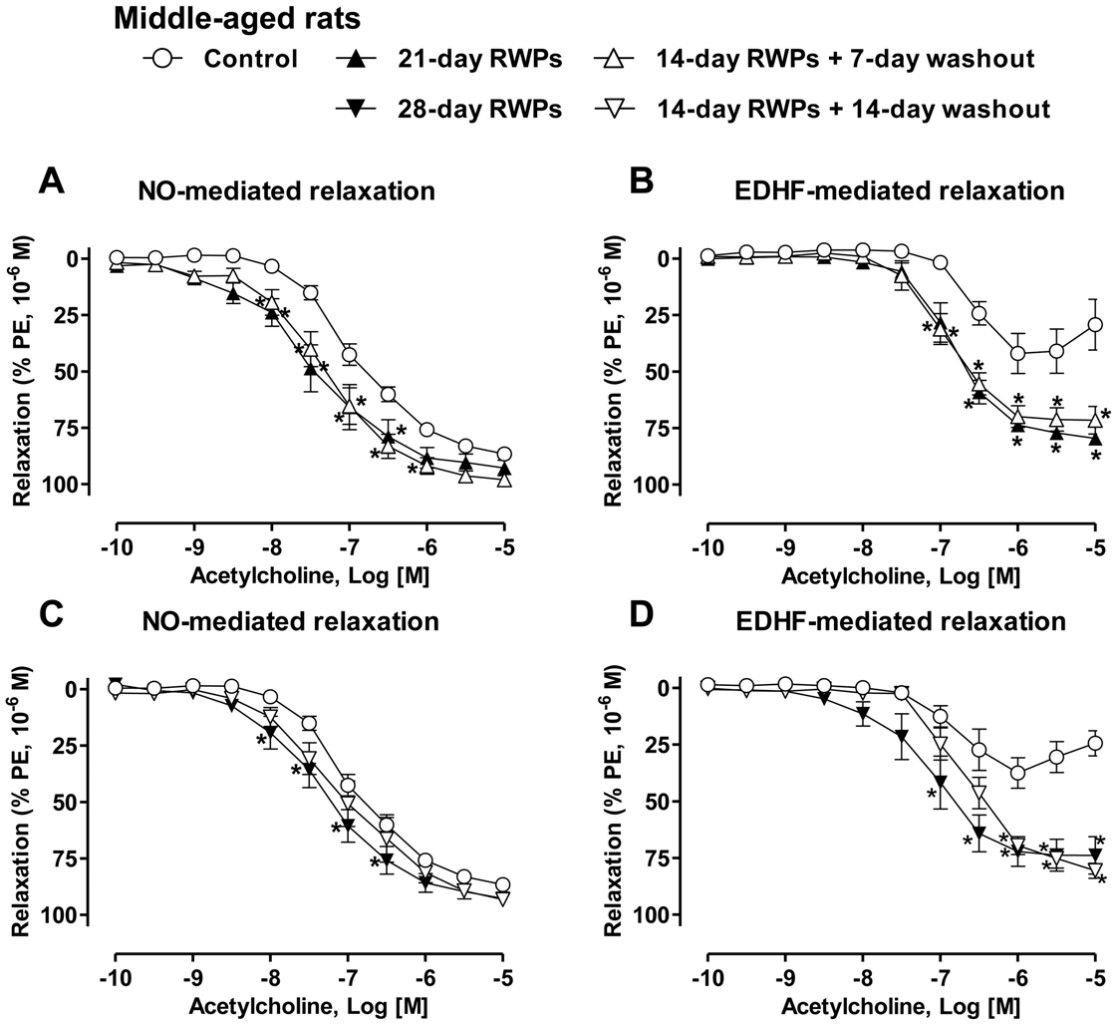
The RWPs-induced Improvement of endothelial dysfunction in the mesenteric artery persists after 7 and 14-day washout periods. Middle-aged rats (46-week old) received RWPs (100 mg/kg/day) in the drinking water for either 21 days or 14 days followed by a 7-day washout period (a, b), and for 28 days or 14 days followed by a 14-day washout period (c, d). NO-mediated relaxations were determined in rings contracted with phenylephrine (1 µM) in the presence of indomethacin (10 µM) and charybdotoxin (CTX, 100 nM) plus apamin (APA, 100 nM) to inhibit the participation of prostanoids and EDHF, respectively (a, c). EDHF-mediated relaxations were recorded in the presence of indomethacin (10 µM) and N^ω^-nitro-L-arginine (L-NA, 300 µM) to rule out the participation of prostanoids and NO, respectively (b, d). Results are shown as means ± SEM of 5 to 6 rats. *P<0.05 indicates a significant difference versus the middle-aged rat control group.

In order to determine whether the protective effect of a 14-day treatment of middle-aged rats with RWPs (100 mg/kg/day) on the endothelial function persists after stopping their intake, rats were treated with RWPs for 14 days followed by a 7- or 14-day intake of solvent and then the reactivity of the mesenteric artery was determined. Intake of RWPs for either 21 or 28 days was associated with a slight but significant improvement of the acetylcholine-induced NO-mediated relaxation and a marked improvement of the EDHF-mediated relaxation (Figure 3). The protective effect of the 14-day RWPs treatment on the NO component was also observed one but not two weeks after stopping the intake of RWPs whereas that on the EDHF component persisted thereafter for two weeks (Figure 3). Thus, these data indicate that the protective effect of the RWPs treatment on the endothelial function is a sustained event, which persists, at least, two weeks after stopping their intake.

### RWPs improve aging-related down-regulation of the expression of eNOS, SK_Ca_ and IK_Ca_, in the mesenteric artery of middle-aged rats

**Figure 4:**
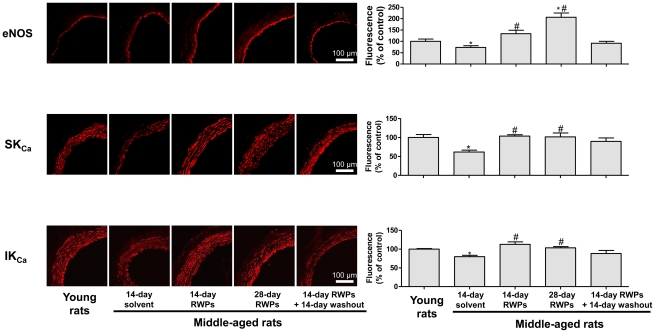
RWPs improve aging-related down-regulation of eNOS, SK_Ca_ and IK_Ca_ expression in the mesenteric artery. The expression level of eNOS, SK_Ca_ and IK_Ca_ (small- and intermediate-conductance Ca^2+^-activated K+ channel, respectively) was determined in mesenteric artery sections using purified polyclonal antibodies and a fluorescence-tagged secondary antibody by confocal microscopy. Left panels show representative immunofluorescence staining and right panels corresponding cumulative data. Results are shown as mean ± SEM of 4 different rats. *P<0.05 indicates a significant difference versus the young rat control group and ^#^ *P*<0.05 versus the middle-aged rat control group. All micrographs were taken at the same magnification.

Immunohistochemical staining of eNOS in mesenteric artery sections indicated a slightly but significantly reduced staining in middle-aged rats compared to young rats, an effect which was improved by intake of RWPs for either 2 or 4 weeks but not after a 2-week RWPs treatment period followed by a 2-week washout period (Figure 4).

Since blunted EDHF-mediated relaxations have been associated with a reduced expression of SK_Ca_ and IK_Ca_ [24], the expression level of both SK_Ca_ and IK_Ca_ was assessed in mesenteric artery sections by immunohistochemical staining. Strong SK_Ca_ and IK_Ca_ fluorescence signals were observed throughout the arterial wall in young rats (Figure 4). Both the SK_Ca_ and IK_Ca_ fluorescence signals were significantly reduced in the mesenteric artery from middle-aged compared to young rats (Figure 4). Chronic intake of RWPs for either 2 or 4 weeks improved the expression level of SK_Ca_ and IK_Ca_ in the mesenteric artery of middle-aged rats whereas no such effect was observed following a 2-week RWPs treatment period followed by a 2-week washout period (Figure 4).

### RWPs reduce oxidative stress and the expression of NADPH oxidase subunits nox1 and p22phox in the mesenteric artery of middle-aged rats

**Figure 5:**
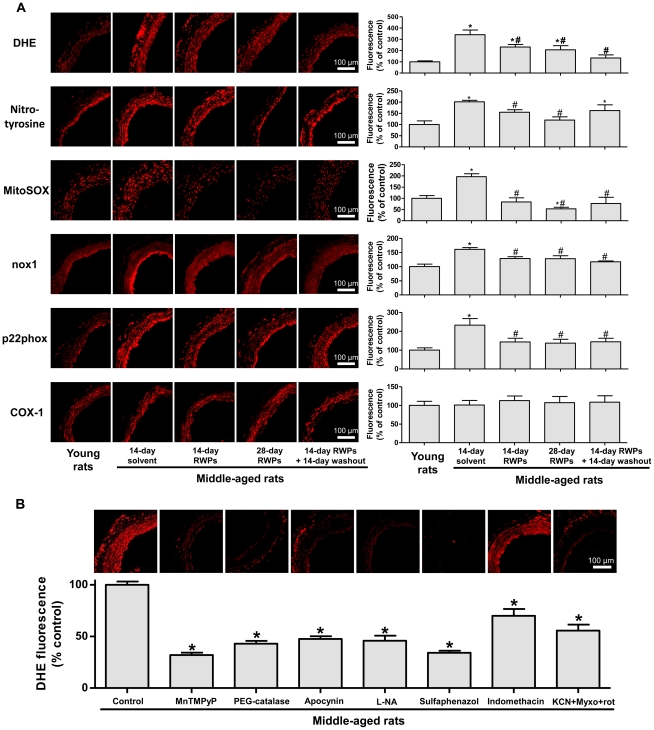
RWPs treatment reduces oxidative stress in the mesenteric artery of middle-aged rats: several sources are involved. Oxidative stress in mesenteric artery sections was determined using the redox-sensitive fluorescent dye dihydroethidine (DHE), nitrotyrosine immunohistochemical staining and the mitochondrial redox-sensitive dye, MitoSOX. In addition, immunohistochemical staining of the NADPH oxidase subunits nox1 and p22phox, and COX-1 is also shown. a) Left panels show representative photographs; right panels corresponding cumulative data. b) Mesenteric artery sections from middle-aged rats were exposed either to MnTMPyP (membrane-permeant superoxide dismutase mimetic), PEG-catalase (membrane-permeant catalase), apocynin (NADPH oxidase inhibitor), L-NA (NO synthase inhibitor), sulfaphenazol (cytochrome P450 inhibitor), indomethacin (cyclooxygenase inhibitor) or inhibitors of the mitochondrial respiration chain (KCN, myxothiazol, and rotenone) for 30 min before DHE staining. Upper panel represents ethidium staining; lower panel corresponding cumulative data. Results are shown as mean ± SEM of 4 different rats. *P<0.05 indicates a significant difference versus the young rat control group (a) and the middle-aged rat control group (b), and ^#^ *P*<0.05 versus the middle-aged rat control group. All micrographs were taken at the same magnification.

Since aging is associated with increased vascular oxidative stress, and oxidative stress with a reduced expression of SK_Ca_ and IK_Ca_ in the rat mesenteric artery [14], [24], the possibility that the RWPs treatment improves the level of oxidative stress in the mesenteric artery of middle-aged rats was assessed using the redox-sensitive fluorescent probe dihydroethidine (DHE). The DHE fluorescence signal was markedly increased throughout the arterial wall in middle-aged compared to young rats (Figure 5a). Intake of RWPs for 2 weeks, 4 weeks or 2 weeks followed by a 2-week washout period was associated with a significant reduction in the DHE fluorescence signal in the mesenteric artery of middle-aged rats (Figure 5a).

In order to determine the nature and source of ROS, mesenteric artery sections from middle-aged rats were treated with different inhibitors before DHE staining. The DHE fluorescence signal in mesenteric artery sections of 46-week old rats was markedly reduced by membrane-permeant analogues of superoxide dismutase (MnTMPyP) and catalase (PEG-catalase) indicating the involvement of both superoxide anions and hydrogen peroxide (Figure 5b). In addition, the increased DHE fluorescence signal was also markedly reduced by apocynin (a NADPH oxidase inhibitor), L-NA (an endothelial NO synthase inhibitor), sulfaphenazol (a cytochrome P450 inhibitor), indomethacin (a cyclooxygenase inhibitor) and by a combination of inhibitors of the mitochondrial respiration chain (KCN, myxothiazol, and rotenone) indicating the involvement of NADPH oxidase, uncoupled eNOS, cytochrome P450, cyclooxygenases and the mitochondrial respiration chain (Figure 5b). In addition, the level of oxidative stress in the mesenteric artery was also assessed by nitrotyrosine staining, an indicator of the formation of peroxynitrites. As indicated in Figure 5a, an increased signal was observed in the mesenteric artery of middle-aged rats compared to young rats, an effect which was prevented by the RWPs treatment for either 2 or 4 weeks (Figure 5a). Moreover, the level of mitochondrial oxidative stress was assessed using the redox-sensitive dye MitoSOX. As indicated in Figure 5a, an increased staining was observed in the mesenteric artery of middle-aged rats compared to young rats, and this effect was prevented by the intake of RWPs for either 2, 4 or 2 weeks followed by a 2-week washout period (Figure 5a).

Since the characterization of the enzymatic sources of ROS in the mesenteric artery of middle-aged rats has indicated the involvement of NADPH oxidase and cyclooxygenases, their expression level was determined by immunohistochemical staining. Both the signal for NAPDH oxidase subunits nox1 and p22phox were significantly increased in middle-aged rats compared to young rats, and this effect was prevented by the RWPs treatments (2 weeks, 4 weeks and 2 weeks followed a 2-week washout period; Figure 5a). In contrast, the COX-1 signal was similar in all groups (Figure 5a), and that of COX-2 was barely detectable (data not shown).

### RWPs normalize the aging-related alterations of the angiotenin system

**Figure 6:**
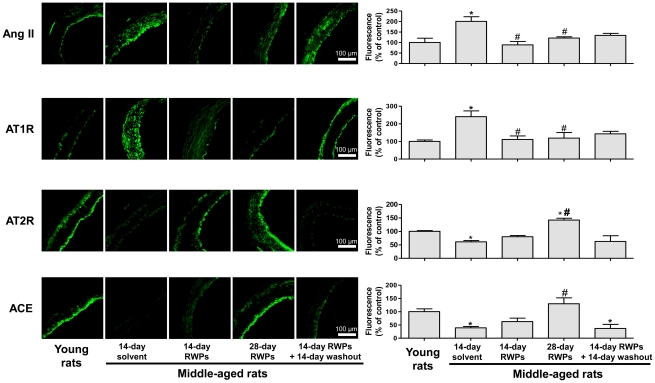
RWPs improve the aging-related over-expression of several components of angiotensin system in the mesenteric artery. The expression level of angiotensin II (Ang II), AT_1_ receptor (AT1R), AT_2_ receptors (AT2R) and angiotensin-converting enzyme (ACE) was determined by immunohistochemical staining. Left panels show representative immunofluorescence staining; right panels corresponding cumulative data. Results are shown as mean ± SEM of 4 different rats. *P<0.05 indicates a significant difference versus the young rat control group and ^#^ *P*<0.05 versus the middle-aged rat control group. All micrographs were taken at the same magnification.

Since previous studies have suggested the involvement of the angiotensin system in the aging-related endothelial dysfunction [10], [17], the expression level of angiotensin II, AT1 receptors, AT2 receptors and angiotensin-converting enzyme (ACE) was assessed by immunohistochemical staining in the mesenteric artery of young and middle-aged rats. The expression level of angiotensin II and AT1 receptors was predominantly associated with the luminal surface and also, to some extent, the adventitia in the mesenteric artery of young rats (Figure 6). The expression level of both Ang II and AT1 receptors was significantly increased in the mesenteric artery of middle-aged rats (Figure 6). The Ang II fluorescence was associated predominantly with the luminal surface whereas that of AT1 receptors was observed throughout the arterial wall (Figure 6). Strong AT2 receptor and ACE fluorescence signals were observed predominantly at the luminal surface of the mesenteric artery of young rats whereas they were markedly reduced in the mesenteric artery of middle-aged rats (Figure 6).

Intake of RWPs for either 2 or 4 weeks was associated with expression levels of Ang II and AT1 receptors in the mesenteric artery of middle-aged rats similar to those observed in young rats (Figure 6). The RWPs treatment improved also the AT2 receptor and ACE signals but only after a 4-week treatment period (Figure 6). The beneficial effect of the RWPs treatment on Ang II, AT1 receptors, AT2 receptors and ACE levels were not observed after 2 weeks of RWPs intake followed by a 2-week washout period (Figure 6).

## Discussion

The major findings of the present study indicate that established aging-related blunted endothelium-dependent NO- and EDHF-mediated relaxations are improved by oral intake of RWPs; such an effect is observed already 7 days after intake of RWPs and is maintained when the RWPs intake period is extended up to 28 days. Moreover, the beneficial effect of a 14-day intake of RWPs on EDHF and NO components is still observed 7 and/or 14 days after stopping intake of RWPs indicating that the protective effect of RWPs on aging-related endothelial dysfunction is a sustained event. The present findings further indicate that aging is associated with an increased vascular formation of ROS, in particular superoxide anions and hydrogen peroxide, which appears to involve several sources including NADPH oxidase, uncoupled eNOS, cytochrome P450, cyclooxygenases and the mitochondrial respiration chain. Vascular aging is also associated with an increased activation of the angiotensin system as indicated by enhanced immunohistochemical staining of angiotensin II and AT1 receptors in the arterial wall. Since angiotensin II is a strong activator of vascular oxidative stress via AT1 receptor and an inducer of endothelial dysfunction, it is likely that the protective effect of the RWPs treatment on aging-related endothelial dysfunction is due, at least in part, to its ability to normalize the expression of angiotensin II and AT1 receptors in the mesenteric arterial wall of middle-aged rats.

The study of the endothelial function in the mesenteric artery as a function of aging has indicated a major impairment of the EDHF component and also, to some extent, the NO component. Aging-related blunted NO-mediated relaxations have also been observed in the second and the third branch of the superior mesenteric artery [8], the aorta [14], the carotid artery [6], the coronary arterioles [25] and the perfused kidney [26] in the rat. In aging, an impairment of EDHF-mediated relaxations has been observed in the superior mesenteric artery and the perfused kidney of rats [9], [23], [26], and the human gastroepiploic distal artery [27]. Although the aging-related endothelial dysfunction has been consistently observed, great variations in the extent of the impairment of the NO and/or the EDHF components have been reported and have been attributed to the use of different rat strains, arterial types and the difference of age of the old rats [5], [6], [8], [9], [10], [17], [26]. In addition, we have observed a pronounced impairment of the NO component in the rat mesenteric artery of 40-week old Wistar rats in a previous study whereas only a small impairment was observed in the mesenteric artery of 46-week old Wistar rats in the present study [23]. Although surprising such a difference may be due to the fact that the former rats were from our inbred strain whereas the latter ones were bought from a commercial supplier.

The present findings indicate that intake of RWPs (100 mg/kg/day) improved both NO- and EDHF-mediated relaxations in the mesenteric artery of middle-aged rats and that this effect is observed already after a 7-day treatment period and persisted up to a 28-day treatment period. The dose of 100 mg/kg/day RWPs corresponds to a human equivalent dose of ∼1000 mg/day [28]. Our previous study has also indicated that chronic intake of low doses of RWPs (25 or 75 mg/kg/day) by 16 week-old young rats for 24 weeks is associated with improved NO and EDHF-mediated relaxations in the rat mesenteric artery [23]. Thus, RWPs appear to be able to retard the development of an endothelial dysfunction and to improve an established endothelial dysfunction affecting both NO and EDHF related to aging. Since the improvement of the NO component was still observed one week after stopping intake of RWPs and that of the EDHF component persisted up to 2 weeks in middle-aged rats, the beneficial effect of RWPs on the endothelial function appears to be a long lasting event, most likely, due to their ability to accumulate into tissues [29].

The aging-related impairment of the EDHF component seems to be mostly due to a reduced expression of SK_Ca_ and IK_Ca_ channels, which mediate EDHF-dependent hyperpolarization and relaxation in the rat mesenteric artery [3]. A reduced expression level of SK_Ca_ and IK_Ca_ associated to blunted EDHF-mediated relaxations has also been observed in the rat mesenteric artery following angiotensin II-induced hypertension [30] or common bile duct ligation [24], and in the rat cavernous tissue following streptozotocin-induced diabetes [31]. The present findings suggest that the beneficial effect of the RWPs treatment on the EDHF component might be due, at least in part, to their ability to restore the expression of SK_Ca_ and IK_Ca_ to a level similar to that observed in young adults. The RWPs treatment also increased the reduced eNOS fluorescence signal in the mesenteric artery of middle-aged rats; such an effect might contribute to explain the improved NO-mediated relaxation. Lower levels of eNOS expression in old blood vessels have also been observed previously in some studies [25], [32] whereas others reported an increased eNOS expression level [8], [14], [23]. Such a heterogeneous effect might be related to the use of different types of arteries, the difference of age of the old rats, and possibly also to the fact that aged rats were from different suppliers.

Several studies have indicated that vascular oxidative stress plays a major role in aging-related endothelial dysfunction [14],[25],[33]. Indeed, an increased level of superoxide anions [14], [25], [33], and also hydrogen peroxide as shown in the present study, is observed in the aged arterial wall. Moreover, several sources of reactive oxygen species have been involved and, in particular, NADPH oxidase in the rat aorta and mesenteric artery [33], [34], xanthine oxidase in the rat aorta [35], cyclooxygenase-2 in pig pial arteries [36], and uncoupled endothelial NO synthase in the mouse mesenteric artery [37]. The present findings suggest that, besides NADPH oxidase, cyclooxygenases, and uncoupled endothelial NO synthase, cytochrome P450 monoxygenases and the mitochondrial respiration chain contribute also to the increased oxidative stress in the mesenteric artery of middle-aged rats. Superoxide anions are known to reduce the bioavailability of NO both by reacting with NO to form peroxynitrite and by oxidizing tetrahydrobiopterin, an essential co-factor of NO synthase [38], [39]. Moreover, the beneficial effect of the RWPs treatment on aging-related endothelial dysfunction appears to involve their ability to prevent the excessive oxidative stress in the aged arterial wall. The antioxidant effect may due to their ability to directly interact with superoxide anions and other reactive oxygen species such as hydroxy and peroxy radicals [40]. It may also be due to their ability to prevent the aging-related upregulation of the NADPH oxidase subunits nox1 and p22phox, and to improve the mitochondrial formation of ROS as shown in the present study. Previous studies have also shown that RWPs prevent the expression of NADPH oxidase in the aorta subsequently to the infusion of a hypertensive dose of angiotensin II to rats [18]. Moreover, tea polyphenols prevented the expression of NADPH oxidase and up-regulated that of catalase in vascular cells [41], [42].

Although the mechanism underlying the aging-related oxidative stress-mediated endothelial dysfunction remains to be determined, several lines of evidence support a role for the angiotensin system. Indeed, angiotensin II is a potent inducer of endothelial dysfunction and vascular oxidative stress [19], [43]. Moreover, treatment of rats with either an angiotensin-converting enzyme inhibitor or an AT1 receptor antagonist effectively ameliorated endothelial dysfunction, both the NO component and the EDHF component, in aged blood vessels, in part, by decreasing vascular oxidative stress [7], [9], [10], [44]. Moreover, in old AT1-receptor deficient mice, endothelium-dependent relaxations in the basilar artery were normal whereas those in wild-type mice were reduced by about 50% [45]. The present findings also support a role for the angiotensin system in the aging-related endothelial dysfunction since aging was associated with an increased expression level of angiotensin II and AT1 receptors. In addition, aging was also associated with reduced expression of AT2 receptors, which have been shown to stimulate the endothelial formation of NO [46]. Although the source of angiotensin II remains to be clarified, it does not seem to involve the angiotensin-converting enzyme since its expression level appears to be down-regulated in the mesenteric artery of middle-aged rats. The possibility that the local angiotensin system contributes to the increased expression level of angiotensin II in aged blood vessels still remains to be studied. Such a possibility might involve chymases, which have been shown to mediate the high glucose-induced formation of angiotensin II in rat vascular smooth muscle cells [47]. The present findings indicate that the intake of RWPs is able to normalize the expression level of angiotensin II, AT1 receptors, AT2 receptors and angiotensin-converting enzyme in the mesenteric artery of aged rats to a level similar to that observed in young rats. In addition, intake of RWPs by rats starting at week 16 until week 40 prevented also the expression of AT1 receptors in the mesenteric artery [23]. The increased formation of angiotensin II in aged blood vessels most likely contributes, besides oxidative stress, to the down-regulation of SK_Ca_ and IK_Ca_ since a reduced expression level of both channels is observed in the rat mesenteric artery during angiotensin II-induced hypertension [30].

In conclusion, aging is associated with the development of an endothelial dysfunction in the rat mesenteric artery, which affects markedly the EDHF component and also, to some extent, the NO component. Intake of RWPs in the drinking water improves the established aging-related endothelial dysfunction most likely by normalizing the local activation of the angiotensin system and preventing the oxidative stress-mediated impairment of both the NO and the EDHF components.

## Materials and Methods

### Ethics statement

This study conforms to the Guide of Care and the Use of laboratory Animals published by the US National Institutes of Health (NIH publication No. 85-23, revised 1996) and the present protocol was approved by the local ethics committee (Comité Régional d'Éthique en Matière d'Expérimentation Animale, approval AL/01/09/09/05).

### Preparation of red wine polyphenols (RWPs)

RWPs dry powder, obtained from French red wine (Corbières A.O.C., France), was provided by Dr. M. Moutounet (Institut National de la Recherche Agronomique, Montpellier, France) and analyzed by Pr. P.-L. Teissedre (Université de Bordeaux, France). One liter of red wine produced 2.9 g of RWPs, which contained 471 mg/g total phenolic compounds expressed as gallic acid equivalents. The extract contained 8.6 mg/g catechin, 8.7 mg/g epicatechin, dimers (B1, 6.9 mg/g; B2, 8.0 mg/g; B3, 20.7 mg/g; B4, 0.7 mg/g), anthocyanins (malvidin-3-glucoside, 11.7 mg/g; peonidin-3-glucoside, 0.66 mg/g; cyanidin-3-glucoside, 0.06 mg/g), and phenolic acids (gallic acid, 5.0 mg/g; caffeic acid, 2.5 mg/g; caftaric acid, 12.5 mg/g).

### *In vivo* treatment of rats

Food and water were given *ad libitum*. The study was conducted in 12 young (16 weeks), 6 mature-adult (25 weeks) and 48 middle-aged (46 weeks) male *Wistar* rats. Rats were divided into groups of 6 rats receiving either solvent (3% ethanol v/v) or red wine polyphenols (100 mg/kg/day) in the drinking water for different periods as indicated. Before sacrifice, rats were anaesthetized with pentobarbital (50 mg/kg, intraperitoneally). After excision, the mesenteric artery was placed in Krebs bicarbonate solution for the subsequent determination of vascular reactivity using organ chambers, immunohistochemical staining and measurement of vascular oxidative stress.

### Vascular reactivity studies

The main superior mesenteric artery was cleaned of connective tissue and cut into rings (2–3 mm in length). This type of blood vessel has been selected due to the fact that endothelium-dependent relaxations involve both NO and EDHF as in human coronary arteries, which are the major site for the development of cardiovascular diseases such as atherosclerosis [48]. In some preparations, the endothelium was removed by rubbing the intimal surface of rings with a pair of forceps. Rings were suspended in organ baths containing oxygenated (95% O_2_, 5% CO_2_) Krebs bicarbonate solution (mM: NaCl 119, KCl 4.7, KH_2_PO_4_ 1.18, MgSO_4_ 1.18, CaCl_2_ 1.25, NaHCO_3_ 25 and d-glucose 11, pH 7.4, 37°C) for the determination of changes in isometric tension. The rings were stretched step by step until an optimal resting tension of 1 g was reached and then allowed to equilibrate for at least 60 min. After the equilibration period, the rings were exposed to Krebs bicarbonate solution containing a high concentration of potassium (80 mM) until reproducible contractile responses were obtained. After washing with Krebs bicarbonate solution, the rings were precontracted with phenylephrine (PE, 1 µM) to about 80% of the maximal contraction by high K^+^ solution before addition of acetylcholine (ACh, 1 µM) to check the presence of a functional endothelium. After washout and a further 30-min equilibration period, rings were again contracted with PE before the application of increasing concentrations of either ACh, sodium nitroprusside (an exogenous NO donor) or levcromakalim (an ATP-sensitive K^+^ channel opener) to construct concentration-response curves. Sodium nitroprusside- and levcromakalim-induced relaxations were examined in endothelium-denuded rings of mesenteric artery. In some experiments, rings were exposed to an inhibitor for 30 min before contraction with PE. The NO-mediated component of relaxation was determined in the presence of indomethacin (10 µM) and charybdotoxin (CTX, 100 nM) plus apamin (APA, 100 nM) to inhibit the participation of prostanoids and EDHF, respectively. The EDHF-mediated component of relaxation was determined in the presence of indomethacin (10 µM) and N^ω^-nitro-L-arginine (L-NA, 300 µM) to inhibit the formation of prostanoids and NO, respectively. Relaxations were expressed as percentage of the contraction induced by PE.

### Immunohistochemical determination of target proteins in the mesenteric arterial wall

Segments of the main mesenteric artery were removed, embedded in OCT compound (Tissue-Tek, Sakura Finetek) and snap-frozen in liquid nitrogen. Frozen arteries were cryosectionned at 14 µm. Sections were air-dried for 15 min and stored at −80°C until use. Sections were first fixed with paraformaldehyde at 4%, washed and treated with 10% milk or 5% goat serum in PBS containing 0.1% Triton ×100 for 1 h at room temperature to block non-specific binding. Sections were then incubated overnight at 4°C with an antibody directed against either eNOS (1/100), calcium-dependent potassium channels (SK_Ca_, IK_Ca_, 1/200), angiotensin II (1/500), AT1 receptor (1/400), AT2 receptor (1/400), angiotensin-converting enzyme (1/200), nitrotyrosine (1/200), nox1 (1/200), p22phox (1/200), cyclooxygenase-1 or -2 (1/200), and nitrotyrosine (1/100). Sections were then washed with PBS, incubated with the secondary antibody (Alexa 488-conjugated goat anti-rabbit IgG or Alexa 637-conjugated goat anti-rabbit) diluted (1/400) in the same buffer for 2 h at room temperature in the dark, and washed before being mounted in Vectashield (mounting medium for fluorescence, Vector Laboratories, Inc. Burlingame, CA 94010) and cover-slipped. For negative controls, primary antibodies were omitted. The samples were observed using a confocal laser-scanning microscope (Leica SP2 UV DM IRBE) with 40× magnification lens. Quantification of proteins levels was performed using Image J 1,4 2q software (National Institutes of Health, USA).

### Determination of vascular and mitochondrial ROS formation

The redox-sensitive fluorescent dye dihydroethidine was used to evaluate *in situ* formation of reactive oxygen species (ROS) following the method previously described by Sarr *et al.* [18]. Mesenteric arterial rings (3 to 4 mm length) were embedded in OCT compound (Tissue-Tek), and frozen in a nitrogen bath for cryostat sections. Dihydroethidine (2.5 µM, Sigma) was applied onto 25 µm unfixed cryosections of mesenteric arteries for 30 min at 37°C in a light-protected humidified chamber to determine the *in situ* ROS formation. To determine the nature and the source of ROS, sections were incubated with either MnTMPyP (membrane-permeant superoxide dismutase mimetic, 100 µM), polyethylene glycol-catalase (membrane-permeant catalase, 500 UI/ml), L-NA (NO synthase inhibitor, 300 µM), apocynin (NADPH oxidase inhibitor, 300 µM), sulfaphenazol (cytochrome P450 inhibitor, 100 µM), indomethacin (cyclooxygenase inhibitor, 10 µM) or inhibitors of the mitochondrial respiration chain (myxothiazol, 0.5 µM+rotenone, 1 µM+KCN, 1 µM) for 30 min at 37°C before addition of dihydroethidine. MitoSox (Invitrogen, Carlsbad, CA), a mitochondrion-specific hydroethidine-derivative fluorescent dye, was used to assess mitochondrial ROS *in situ*. Briefly, 14 µm unfixed cryosections of mesenteric arteries were incubated with MitoSox (5 µM at 37°C for 60 min) in a light-protected humidified chamber. Sections were then washed three times, mounted in Vectashield and cover-slipped. Images were obtained with a Leica SP2 UV DM IRBE laser scanning confocal microscope. Quantification of staining levels was performed using Image J 1,42q software.

### Materials

Antibodies were purchased as indicated: mouse anti-eNOS (BD Biosciences Pharmingen, San Diego, California, USA), anti-KCa3.1 (intermediate conductance Ca^2+^-activated K^+^ channel 4, IKCa) and anti-KCa2.3 (small conductance Ca^2+^-activated K^+^ channel 3, SKCa) (Alomone Labs, Jerusalem, Israel), ACE antibody (Abbiotec, San Diego, CA, USA), rabbit anti-angiotensin II (Peninsula laboratories, San Carlos, CA, USA), rabbit anti-AT1 receptor (Santa Cruz Biotechnology), rabbit anti-AT2 receptor (Santa Cruz Biotechnology), mouse anti-nitrotyrosine (United States Biological), mouse anti-nox1 (gp91^phox^) (BD Biosciences Pharmingen, San Diego, California, USA), rabbit anti-p22phox (Santa Cruz Biotechnology), COX-1 Monoclonal Antibody, COX-2 polyclonal antibody (Cayman chemical company, Michigan-USA). Alexa fluor-488 or 637 labelled goat anti-rabbit IgG (Invitrogen, Molecular Probes). Apamin and charybdotoxin were obtained from Latoxan (Valence, France) and N^ω^-nitro-L-arginine, indomethacin, acetylcholine, sodium nitroprusside, levcromakalim from Sigma-Aldrich.

### Statistical analysis

Data are presented as mean ± SEM. of n experiments. Mean values were compared by ANOVA followed by the Bonferroni post-hoc test to identify significant difference between treatments, using GraphPad Prism (version 5 for Microsoft windows. GraphPad software, Inc, San Diego, CA, USA). The difference was considered to be significant when the *P* value was less than 0.05.
